# Synovial inflammation plays a greater role in post-traumatic osteoarthritis compared to idiopathic osteoarthritis in the Hartley guinea pig knee

**DOI:** 10.1186/s12891-017-1913-6

**Published:** 2017-12-29

**Authors:** Nathan P. Thomas, Wesley J. Wu, Braden C. Fleming, Fangyuan Wei, Qian Chen, Lei Wei

**Affiliations:** 1 Department of Orthopaedics, Warren Alpert Medical School of Brown University/RIH, CORO West, Suite 402H, 1 Hoppin Street, Providence, RI 02903 USA; 2 Foot & Ankle Orthopaedic Surgery Center, Beijing Tongren Hospital, Capital Medical University, Beijing, China; 3 Department of Orthopaedics, the Second Hospital of Shanxi Medical University, Shanxi Key Lab of Bone and Soft Tissue Injury Repair, Taiyuan, China

## Abstract

**Background:**

The objective of this study was to evaluate the extent of stromal cell-derived factor-1’s (SDF-1) involvement in the pathogenesis of idiopathic versus post-traumatic OA by comparing differences in synovial membrane morphology, SDF-1 synovial fluid (SF) concentrations, and matrix metalloproteinase-13 (MMP-13) SF concentrations.

**Methods:**

Thirty-six 3-month-old Hartley guinea pigs were obtained and divided into 6 groups. Upon sacrifice, India Ink staining was used to evaluate gross morphology, Safranin O/Fast green staining was used to assess cartilage damage, H/E staining was employed to visualize the synovium, and SF samples were obtained for biochemical analyses. Sandwich ELISA was used to quantify the SF concentrations of SDF-1 and MMP-13.

**Results:**

12 month-old, idiopathic OA guinea pigs and 5.5 month-old ACLT animals had comparable cartilage damage when evaluated by the Modified Mankin Score. SDF-1 and MMP-13 concentrations were not statistically different between the two groups. The synovial membrane of the 5.5 month ACLT group had severe synovitis compared to the idiopathic OA group.

**Conclusion:**

In this study, it was found that synovial inflammation, independent of cartilage morphology, SDF-1 concentration, and MMP-13 concentration, was markedly different between idiopathic and post-traumatic OA. These results highlight the differing morphological and biochemical profiles of post-traumatic versus idiopathic osteoarthritis and calls for a more thorough examination of the sole of the synovial membrane in the pathogenesis of post-traumatic osteoarthritis.

## Background

Although many studies utilize the guinea pig model to study both idiopathic and posttraumatic Osteoarthritis, it is unknown if the pathological mechanisms are the same. Even though OA is primarily classified as a non-inflammatory arthritis, biomechanical stresses affecting the articular cartilage and subchondral bone and biochemical changes in the articular cartilage and synovial membrane are important in its pathogenesis and may be linked to low grade inflammation [1]. Mounting research suggests that synovium mediated inflammatory cytokines may mechanistically differentiate idiopathic and post-traumatic OA, in addition to accelerating disease progression in the post-traumatic situation.

In a previous study evaluating biomarker concentrations between idiopathic and posttraumatic OA in Hartley guinea pigs, Wei et al. reported that 12-month-old ACL transected Hartley guinea pigs, those with PTOA, have accelerated articular cartilage damage when compared to age-matched ACL-intact guinea pigs with idiopathic OA [2]. The greater cartilage damage observed in the ACL-transected knees may be due to either an acceleration of idiopathic OA progression or differences in the pathogenesisincluding the associated inflammatory factors.

It was also found that same-age ACL transected animals presented thicker synovial membranes than that of the idiopathic OA animals. Synovial hyperplasia has been associated with cartilage damage in both animal models and human patients. Synoviocytes have been shown to increase synthesis of SDF-1 and different cytokines such as IL-1β and TNFα during inflammation [3–7]. These signaling molecules cause an up-regulation of aggrecanase and matrix metalloproteinase synthesis in chondrocytes and synoviocytes, which digest surrounding cartilage [8]. Therapeutic agents that target the inflammatory cytokines IL-1β and TNF-α have been successful in treating rheumatoid arthritis (RA) and related diseases [9]. Other chemokines, such as SDF-1, may also be involved in OA pathology [10]. Thus, destabilization of the joint via ACL transection may induce greater synovial membrane proliferation compared to a morphologically equivalent idiopathic OA model, which in turn could alter cytokine profiles, and perpetuate the pathogenesis of post-traumatic OA.

SDF-1 has also been found in higher concentrations in ACL transected Hartley guinea pig joints compared to age-matched idiopathic OA joints [2]. Briefly, SDF-1 is an 8KDa chemokine originally isolated from a bone stromal cell line [11]. Through its interaction with CXCR4, it has been shown to stimulate movement of stem cells out of the bone marrow and into circulating blood [12–14]. SDF-1/CXCR interaction also activates calcium, Erk, and p38 MAP kinase signaling pathways in chondrocytes, thereby inducing the release of MMPs, and other proteins required for chemotaxis and other biological processes [15, 16]. Specifically, SDF-1 has been shown to increase the concentration of MMP-3, −9, and −13, thus increasing of the destruction of the ECM proteins [17]. Studies have shown that blocking SDF-1’s receptor, CXCR4 decreases MMP-13 expression in in vitro human chondrocytes [16]. Moreover, it has been shown that synovial cells significantly increase synthesis of SDF-1 during inflammation-induced hypoxia, and that SDF-1 plays a central role in the pathogenesis of murine collagen-induced arthritis by attracting leukocytes to the inflamed joints [18, 19]. Thus, SDF-1 may be an important component in differentiating between idiopathic and post-traumatic OA.

In this study, we tested the hypotheses that post-traumatic (ACL transected) and idiopathic (ACL intact) OA in the Hartley Guinea pig model lead to similar changes in cartilage morphology, but progress via different pathological mechanisms. We assessed pathological mechanisms by evaluating the synovial fluid SDF-1 and MMP-13 concentrations; in addition to quantifying synovial membrane morphology between anatomically similar idiopathic and post-traumatic joints.

## Methods

### Animals

Thirty male Hartley guinea pigs (Elm Labs; Chelmsford, MA) were randomly divided into 5 groups of six animals. Group 1 (*n* = 6) represented the baseline ACL-intact control group (no OA), which were euthanized via CO_2_ at 3-months of age. Group 2 (*n* = 6) represented the idiopathic OA group, which were maintained until 12 months of age. Group 3 (*n* = 6) underwent anterior cruciate ligament transection (ACLT) on the right knee at 3 months and were euthanized at 5.5-months, Group 4 (n = 6) consisted of ACLT animals sacrificed at 6.5-months, and Group 5 (n = 6) consisted of ACLT animals sacrificed at 7.5-month-old. After completion of the first phase of the study, a sham group, group 6 (n = 6), underwent a 1.5 cm sagittal joint capsule incision and was sacrificed at an experimentally determined equivalency point (5.5-months). The sham sacrifice time point was selected to match the time at which the ACLT joints showed similar cartilage damage to the idiopathic OA joints. The contralateral (ACL-intact) knees of the ACL-transected animals served as an additional control (Groups 3, 4, 5; Left knees). The right knee of the sham group (Group 6) was used as a control to account for the biomechanical instability created by the ACLT procedure while the left knee of the sham animals served as a control to gauge the development of post-traumatic OA of the ACLT animals. Three months was determined as an appropriate age to initiate the experiment as guinea pigs have reached skeletal maturity without exhibiting evidence of cartilage damage [20]. All animals were individually housed under standard conditions.

### Surgery

The surgical animals were anesthetized with an intraperitoneal injection of ketamine (40 mg/Kg) and medetomidine (0.5 mg/Kg). The right knee was shaved and prepped with betadine. Animals were placed in the prone position, and a 2 cm midline incision was made over the anterior knee. The skin was mobilized to expose the patellar tendon. A 1.5 cm incision through the joint capsule was made immediately lateral to the patellar tendon. The patella was then everted, and the ACL was incised with the knee in a flexed position. Manual testing of anterior laxity confirmed complete sectioning of the ACL. In sham animals, only a 1.5 cm incision was made penetrating the joint capsule. For all operated groups, the joint capsule and fascia were closed in layers using interrupted 4-0 Vicryl sutures, and skin using 4-0 Nylon sutures. Post-operative analgesia was maintained using buprenorphine hydrochloride (.05 mg/Kg SQ for 3 days). Sutures were removed 10 days post-surgery. Animals were allowed to bear weight on limbs as tolerated, and equal load bearing was noted between limbs within 3 weeks of surgery.

### Macroscopic analysis

The proximal tibias were amputated and immersed in 10% (*v*/v) formalin for at least 72 h. Before histological processing, gross morphological cartilage lesions on tibial condyles were visualized and qualitatively analyzed via India ink staining, in which the cartilage surface is painted, for 15 s, with a 20% (v/v) dilution of blue India ink (Parker, Quink) in phosphate buffered saline containing protease inhibitors [21]. Cartilage samples were qualitatively analyzed for lesion severity and extent.

### Histology

The specimens were then decalcified in 10% EDTA solution and bisected in the coronal plane. They were processed in a Tissue-Tek VIP 1000 tissue processor (Model#4617, Miles, Elkhart, IN) and embedded in a single block of Paraplast X-tra (Fisher, Santa Clara, CA). Blocks were trimmed to expose tissue using a rotary microtome (Model#2030, Reichart-Jung, Austria). Slices were taken from the mid-coronal plane within each condyle and cut into 6 μm thick sections, taken 0 μm, 100 μm, and 200 μm, from the midcoronal line. Sections were then mounted on slides and stained with safranin-O/fast green. The severity of cartilage damage of each joint was assessed using the modified Mankin grading system [20]. Three independent observers scored both the medial and tibial condyle compartments using a digital imaging camera (Nikon microscope E 800). All scoring was done in a random order and in a blinded fashion. The worst scores for each medial and lateral tibial compartment were statistically analyzed. Medial, lateral, and infrapatellar compartments of synovial membrane specimens were dissected, fixed, embedded, cut and stained as described above. Using the Pelletier grading system, the worst synovial scores from each joint were analyzed [22]. All histological imaging utilized the SPOT digital imaging camera (Diagnostic Instruments, Sterling Heights, Michigan).

### Synovial fluid (SF) collection and analysis

Before dissecting the joints, 100 μL of isotonic saline solution was injected intra-articularly, the knee was manually cycled through flexion and extension ten times and then the joint was aspirated. The lavaged synovial fluid was centrifuged at 2000 g for 10 min to remove cells and debris, and then frozen at −80 °C until analysis.

Prior to the SDF-1 and MMP-13 analysis, 20 μL aliquots were treated with 15 U/ml of bovine testicular hyaluronidase for 10 min at 37 °C to reduce viscosity. SDF-1 and MMP-13 concentrations in the SF samples were measured in duplicate. Commercially available double-antibody sandwich enzyme-linked immunosorbent assays (ELISA) were used to detect, SDF-1α (DSA00, R&D, Minneapolis, MN) and MMP-13 (F13 M00, R&D Systems, Minneapolis, MN) in the synovial lavages following the manufacturer’s instructions.

### Statistics

Analyses of variance (ANOVA) for repeated measures were used to compare the histological measurements of cartilage damage and synovial membrane inflammation between the six groups. Similar analyses were performed to evaluate the concentrations of SDF-1 and MMP-13. The different weights of the guinea pigs between groups were considered using analysis of covariance (ANCOVA). A two-way mixed absolute interclass reliability coefficient (ICC) was calculated to evaluate the reliability of both the Mankin and Pelletier scoring systems. Follow-up pair-wise comparisons between multiple experimental groups were carried out with orthogonal contrasts using the Tukey’s test (α = .05) and test of homogeneity. Adjusted *p*-values were reported to account for the multiple comparisons. Prior to analysis, normality was confirmed with the Shapiro-Wilk test. Statistics were performed on SPSS software (SPSS Inc., Chicago, Illinois).

## Results

### Gross morphology

**Fig. 1:**
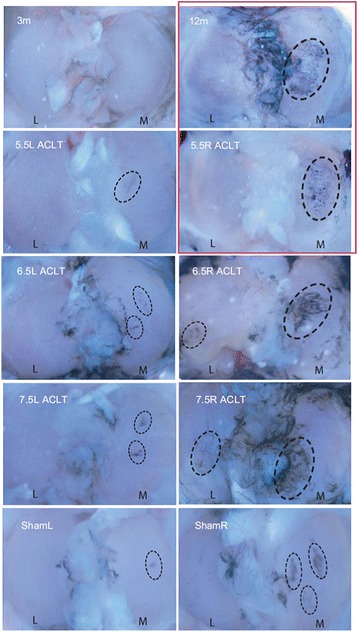
India ink staining of 3-month, 12-month, 5.5-ACLT, 6.5-ACLT, 7.5-ACLT, and Sham tibial joints. The right tibia of the 5.5-ACLT joint looked most similar to that of the 12-month primary OA joint (red box). Size and intensity of the staining marked severity of the joint (dashed circles). (Original magnification, × 10)

India ink staining revealed the presence of cartilage lesions in all ACLT (post-traumatic OA) and the 12-mo ACL intact group (idiopathic OA). There was a progressive increase in number and size of lesions with age in the ACLT group animals that first presented in the medial condyle, and then became more prominent on the lateral side**.** The quality, size, and intensity of the lesions between the primary OA joints (12-months) and the youngest ACLT joints (5.5-ACLT) were similar, as seen in Fig. 1. The baseline control (3-months) had no detectable lesions, while the contralateral controls for the ACLT joints and sham group had moderate focal lesions in random locations.

### Modified Mankin scoring

**Fig. 2:**
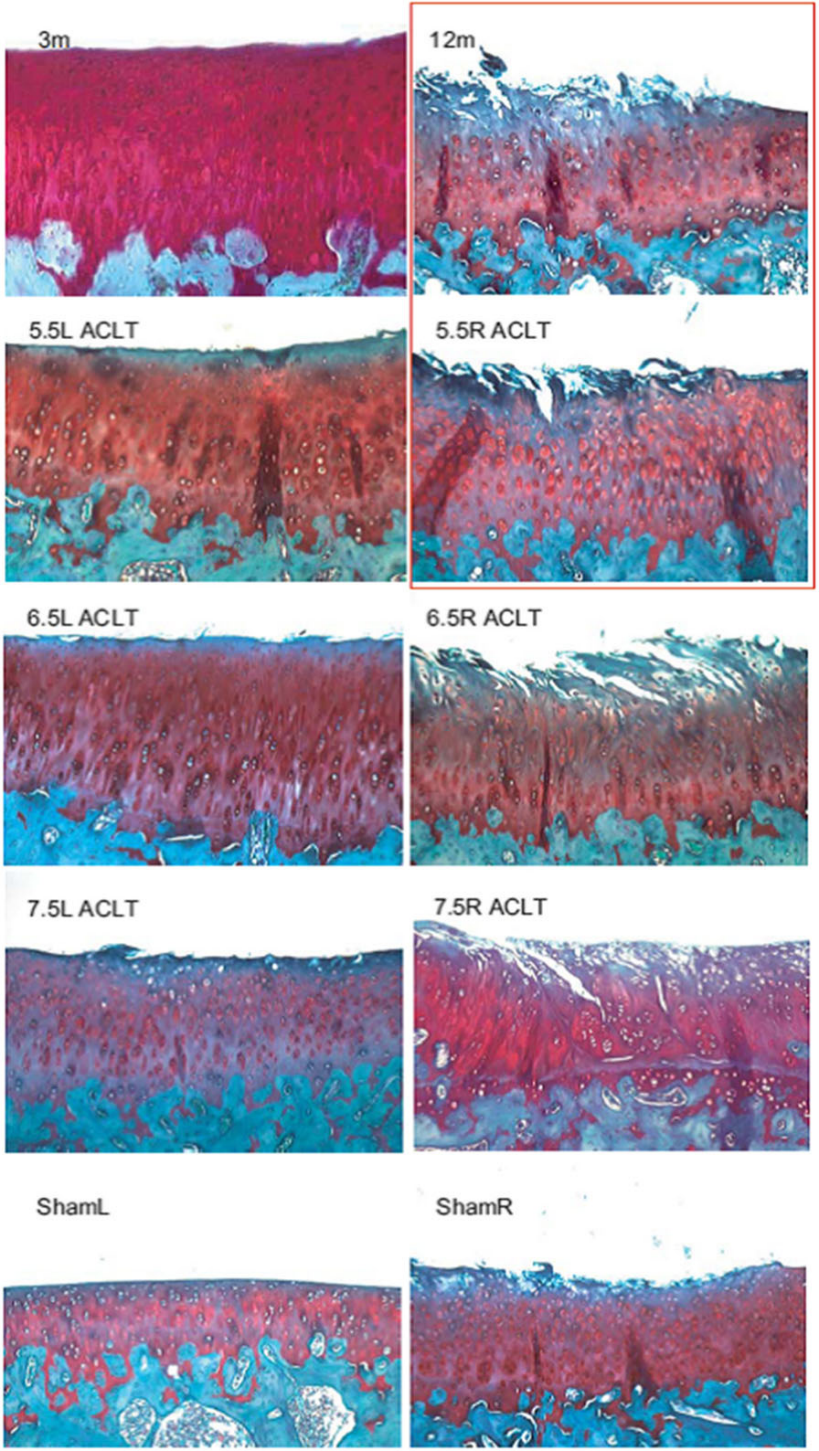
Safranin O/Fast Green staining of representative, median scoring, 3-month, 12-month, 5.5-ACLT, 6.5-ACLT, 7.5-ACLT, and Sham medial tibial condyles. The right medial tibia of the 5.5-ACLT month joint looked most similar to that of the 12 month primary OA joint (**red box**). (Original magnification, × 100)

Safranin-O/fast green stain showed severe cartilage damage in the 7.5-ACLT group, as exemplified by structural clefts into to the middle and deep layer with severe loss of proteoglycan in the superficial, middle, and deep zones of the articular cartilage (Fig. 2). Cell clustering and hypocellularity was noticeable. Histologically severe OA was also evident in the 6.5-ACLT group, which had significant cartilage damage and loss of proteoglycan staining throughout the middle zone. The 12 month-old idiopathic OA joints had moderate OA lesions breaching the superficial and middle layers, very similar to the right 5.5 month-old ACLT joints. The ACLT contralateral limbs had a notable amount of cartilage damage, which progressed by age but were minimal compared to the operated knee. The non-operated knee of the sham animals had virtually no cartilage damage although minimal proteoglycan staining loss differentiated it from the 3 month-old, baseline animals. Surprising, the sham right knee showed notable cartilage damage penetrating throughout the superficial layer and encroaching upon the middle zone in some samples.

**Fig. 3:**
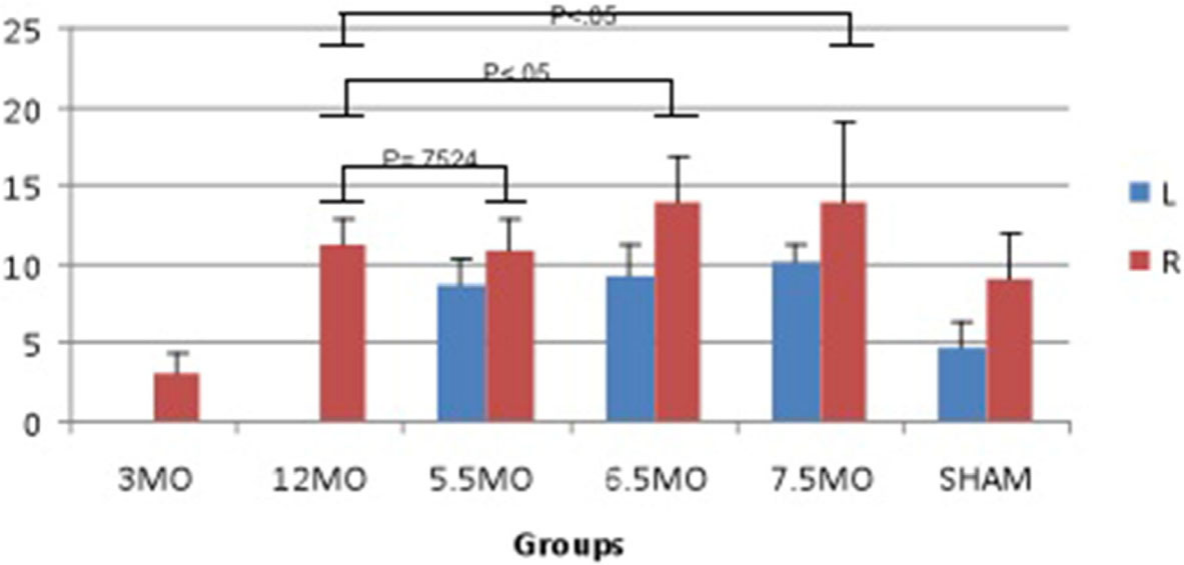
Modified Mankin scores of 3-month baseline (3MO), 12-month primary OA (12MO), 5.5-ACLT, 6.5-ACLT, 7.5-ACLT, and Sham (SHAM) right and left tibial joints. The right knees of the 12-month animals and 5.5-month-old animals were most similar (*p* = .7524), and the 12-month primary OA guinea pigs were statistically significant from the other ACLT groups (P_6.5ACLT_ = .0425, P_7.5ACLT_ = .0389). Error bars represent 1 standard deviation

There was no statistically significant difference in the modified Mankin score between the right knees of the 12-month idiopathic OA group and the 5.5-ACLT group (*P* = .7524), suggesting that these two groups have nearly equivelent cartilage damage (Fig. 3). The cartilage damage scores in both the 6.5-ACLT and 7.5-ACLT animals were significantly greater than that of the 12-month idiopathic OA joints (*p* = .0425 and *p* = .0389, respectively). Non-operated sham knee scores were not significantly elevated compared to 3 month baseline control (*p* = .166), while the right knee exhibited significantly more damage and was comparable to the 5.5-ACLT joint (*p* < .001 and *p* = .0521, respectively). Based on the equivalency of the idiopathic and ACLT group, sham treated animals were sacrificed at 5.5 months of age.

All the ACLT joints produced significantly higher modified Mankin scores compared to the contralateral control leg (P_5.5_ = 0.00133, P_6.5_ = .00301, P_7.5_ < .001). However, the contralateral control knees were significantly more damaged than the 3 month baseline control (group-score interaction: *P* = .004). Inter-rater reliability of the modified Mankin scores was excellent (ICC = .948, 95% CI .914 - .970).

### Pelletier scoring

**Fig. 4:**
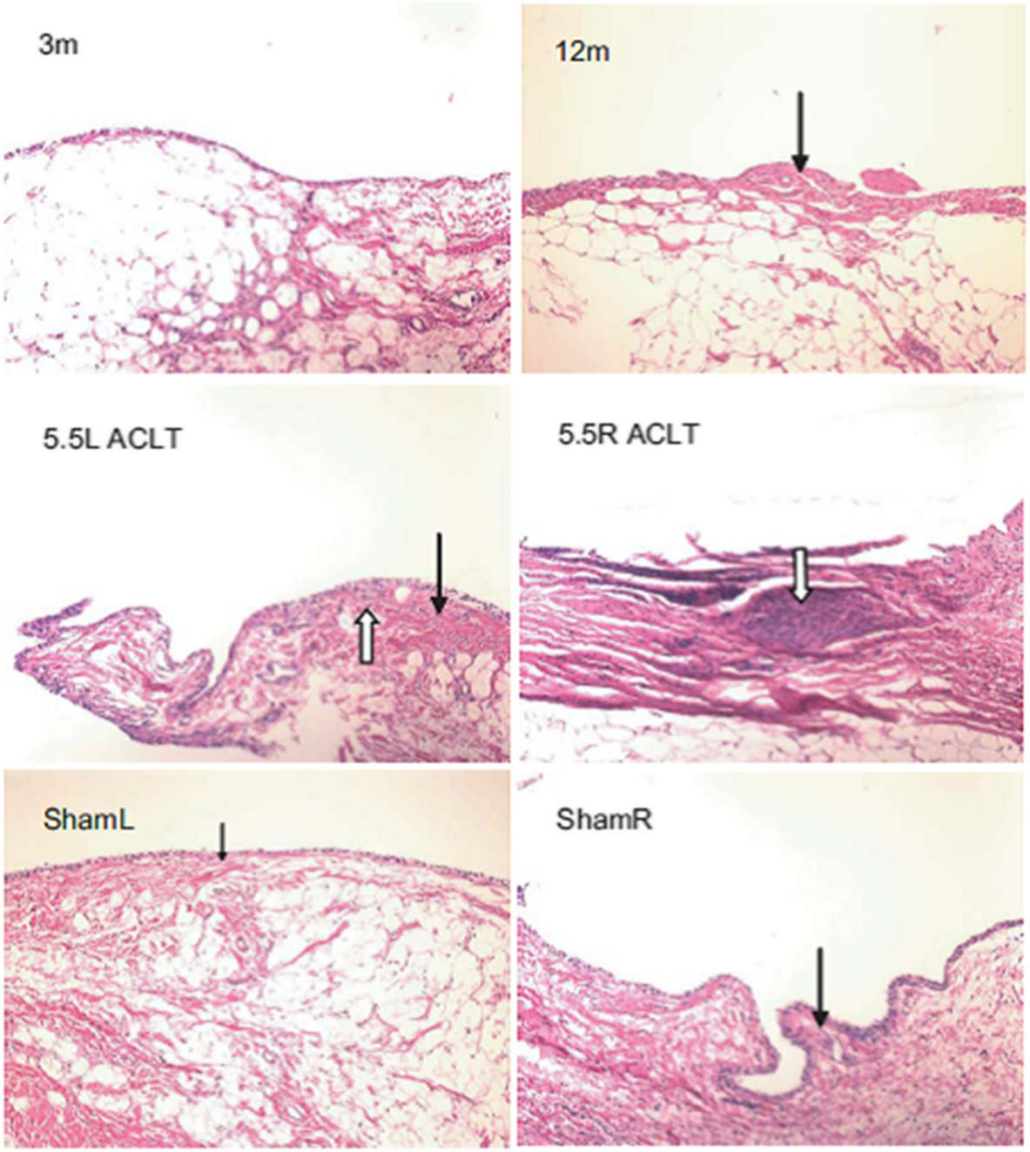
Hematoxylin/Eosin staining of representative 3-month, 12-month, 5.5-month ACLT Sham right and left tibial joints. Normal synovium is depicted with subintimal capillaries and 1-2 cell layers of synoviocytes in the 3-month joint. Surface fibrin is shown in the 12-month synovium (**black arrow**). Synovial hyperplasia is seen in the **white arrow** in the left 5.5 month joint as well as increased fibrosis of the subintimal layer (**black arrow**). The right 5.5-month joint has marked synovitis as pointed (**white arrow**) with leukocyte infiltration. The left sham knee has beginnings of fibrosis (**black arrow**), but no hyperplasia, and the right sham knee has scattered villous hyperplasia (**black arrow**). (Original magnification, × 100)

Differences in the synovial membrane scores were observed between treatment groups (Fig. 4). Surface fibrosis was noted for all synovium samples except those from the 3-month baseline control. Marked synovial hyperplasia was seen in all of the ACLT joints. Villous hyperplasia was seen in the sham right knee, both the right and left 12 month idiopathic OA joints and the 5.5-ACLT joints. The contralateral sham knee had mostly scattered villi. Monocyte infiltration was observed only in the ACLT joints at all three time points.

Pelletier scoring revealed that the 12-month idiopathic OA joints and the 5.5-ACLT joints were statistically similar (*p* < .001). 12-month idiopathic OA joints had a 4-times higher Pelletier score compared to both left and right sham joints, in addition to contralateral controls (group-score interaction: *p* = .00223). The score in the sham operated joint was 9-times higher when compared to the contralateral joint and the 5.5-ACLT unoperated knee (group-interaction *p* = .00574). The contralateral joints (5.5-ACLT-left, Sham-left) were not significantly different (*p* = .209) from each other. No correlation was seen between synovitis scores and modified Mankin scores (r^2^ = .141). The interclass reliability of the Pelletier score was excellent (ICC = .984, 95% CI .953-.995).

### Synovial fluid concentrations of SDF-1 & MMP-13

**Fig. 5:**
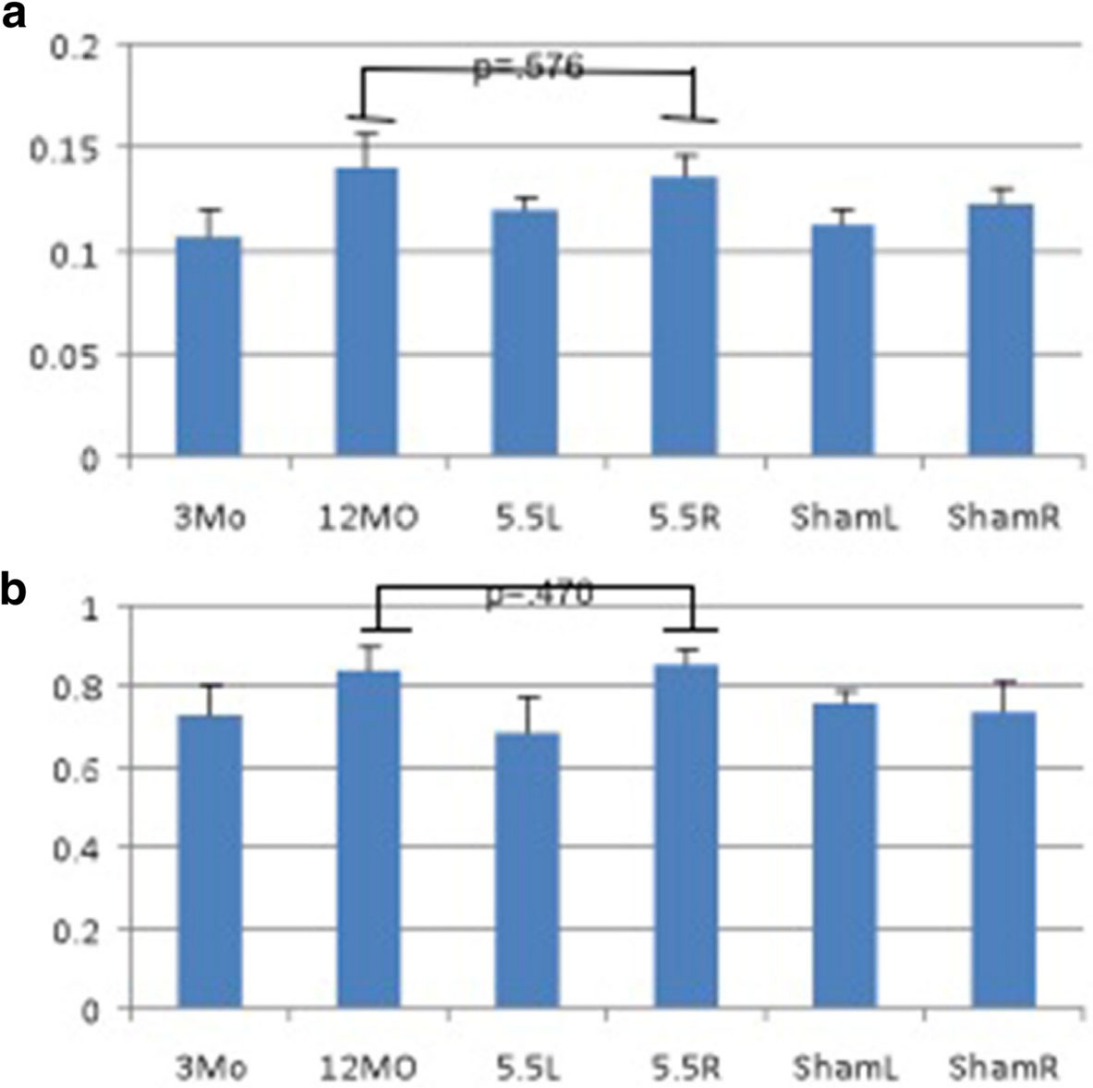
**a** Graph of mean SDF-1 SF concentrations (ng/mL) in 3-month baseline control (3MO), 12-month primary OA (12MO), 5.5-ACLT (5.5R, 5.5 L), and Sham (ShamR, ShamL) synovial fluid. The experimental ACLT group did not have a significantly different SDF-1 concentration compared with that of the 12-month (*p* = .576). **b** Similarly, the experimental ACLT group and the 12-month primary OA SDF-1 had statistically similar MMP-13 synovial fluid concentrations (*p* = .470). Error bars represent one standard deviation

All three ACLT groups and the 12-month idiopathic OA joints showed greater SDF-1 concentrations in comparison to the 3-month baseline control, and sham groups (group-concentration interaction: *p* = .00221) (Fig. 5a). Additionally, in all three of the ACLT and the 12-month idiopathic OA groups, the SDF-1 levels were not significantly different from each other (*p* = .576). The SDF-1 concentrations in the ACLT groups were also significantly different from both the sham and baseline controls (group-concentration interaction: *p* = .019). The left, unoperated, sham joints and 3-month controls were similar (*p* = .226). The baseline control joints were significantly different from both the 5.5-ACLT unoperated joint and the sham operated joint (group-concentration interaction: *p* = .002). SDF-1 synovial fluid concentrations were moderately correlated with the modified Mankin Score (r^2^ = .592) but did not correlate with the synovial membrane score (r^2^ = .195).

Similarly, all ACLT and 12-month idiopathic OA joints showed significantly higher MMP-13 synovial fluid concentrations in comparison to both the baseline control and sham groups (group-concentration interaction: *p* = .000129) (Fig. 5b). However, the synovial fluid MMP-13 levels were not significantly different (*p* = .470) between the ACLT knees and the idiopathic OA knees. The 3-month baseline control, sham joints, and the ACLT synovial fluid MMP-13 concentrations were not significantly different (group-concentration interaction: *p* = .357).

## Discussion

Differentiating the pathophysiology of idiopathic and post-traumatic OA may provide important data regarding the development of therapeutic strategies for different types of OA. The data obtained in this study suggest that the mechanism of disease progression with and without traumatic injury may be different. We found that synovial inflammation, which was independent of SDF-1 and MMP-13, was markedly different between idiopathic and post-traumatic OA. We determined that the cartilage damage seen at 12 months with idiopathic OA was histologically equivalent to that seen at 5.5 months in ACLT animals with post-traumatic OA as indicated by the modified Mankin scores. These findings were also supported by the gross and histological morphology assessments (Figs. 1 and 2).

As hypothesized, histological differences were noted for synovitis in all experimental groups (Fig. 4). The association between OA and synovitis is not fully understood, though previous studies suggest that synovitis is involved. Fernandez-Madrid et al. reported that 73% of OA patients have synovitis, and that the presence of synovitis in early OA may predict the need for joint replacement surgery [23]. It has been hypothesized that synovitis can trigger bone catabolism through the activation of Toll-like receptors (TLRs) and complement cascades [24–27]. Furthermore, synovectomy is an effective surgical method for preventing cartilage destruction and relieving pain in both OA and RA patients [28].

Synovitis has been well characterized in rheumatoid arthritis studies regarding inflammatory cell adhesion and activation, the production of mediators (such as cytokines, chemokines, and growth factors), angiogenesis, joint destruction, fibrosis, and bone resorption [29]. Accordingly, synovitis associated changes were observed microscopically with synovial and villous hyperplasia, surface fibrin deposition, and subintimal fibrosis (Fig. 4) present in both sham left and right, 5.5-ACLT unoperated, 12-month primary OA, and ACLT joints.

In our study, angiogenesis, fibrosis, and leukocyte infiltration were unique to the ACLT joints, suggesting that synovitis is an important mechanism in accelerating cartilage damage. Potential causes of this inflammation could be due to capsulotomy and altered joint biomechanics which in turn may alter cytokine profiles [30]. The combination of both is more likely as the age-matched ACLT joints in our study had both more severe synovitis compared to all other groups and higher modified Mankin scores compared to the sham operated knee. Furthermore, due to the exposure of the joint during capulotomy, the ACL transected animals would be expected to have greater inflammation and synoviocyte activation compared to the sham treated animals.

The idiopathic OA model of this study showed a moderate level of synovitis with notable synovial hyperplasia and fibrosis despite being significantly less severe than the ACLT groups. This result suggests that synovitis may be a part of idiopathic OA pathology but to a lesser degree than in post-traumatic OA following ACL transection. The cause of synovitis in idiopathic OA is unknown, but studies have highlighted potential mechanisms. Because of the lack of a basement membrane and a highly vascularized subintimal layer in the synovial membrane, it is likely that age-related mediators, among others, may induce synovitis [31]. Huebner et al. demonstrated that Hartley guinea pigs, which exhibit idiopathic OA with aging, had greater levels of IL-6 compared with age-matched Strain 13 guinea pigs, which do not exhibit OA [32]. This finding implicates this cytokine in synovitis with idiopathic OA [33]. Synovitis in idiopathic OA may elicit the SDF-1 and MMP-13 signaling pathways, among others, similar to those activated with post-traumatic OA and in combination with age-related changes in proteoglycan turnover, collagen, and vascular changes [34–38].

Further exploration is needed to determine the interactions between the inflammatory mediators and synovitis. Although elevated in relatively-accelerated cartilage damage in our previous study, SDF-1’s induction of cartilage degradation via MMP-13 may not be involved in pathologically differentiating idiopathic and post-traumatic OA, as no statistical significance was observed between the 12-month primary OA guinea pig knees and the 5.5-ACLT knees. There was also a moderate correlation between increases in SDF-1 and modified Mankin scores for all treatment groups (r^2^ = .592), suggesting that SDF-1 may provide a downstream mechanism for both idiopathic and post-traumatic OA. Unfortunately, no other studies have compared SDF-1’s involvement in idiopathic or post-traumatic OA models. Because SDF-1 and MMP-13 do not seem to play a greater role in either idiopathic or post-traumatic OA in guinea pig models, other inflammatory factors may be involved. Elsaid et al. has proposed a possible mechanism of secondary OA pathogenesis via synovial upregulation of IL-1β, TNFα, procathepsin B, and neutrophil elastase, which are capable of being synthesized by the synovium and downregulate lubricin, a chondroprotective and lubricating protein of articular cartilage [39].These cytokines may also regulate chondrogenic production of inflammatory factors, synovial cell overgrowth, and collagenase [40].

Surprisingly, the contralateral unoperated knee in ACL transected animals presented with moderate OA. Compensatory biomechanical processes may cause a shift in knee kinematics, sensitization of chondrocytes, followed by subsequent reorganization and degeneration favoring catabolic over anabolic cellular processes [31]. Cartilage damage in the contralateral joint may also be further exacerbated by systemic inflammatory effects related to OA in the operated knee.

There were some limitations to the study. First, we were only able to obtain a mean volume of 120 μL from each animal, and this amount reduced our ability to run additional ELISAs and other experiments. Many previous studies have used GAG, C2C, and COMP to quantify the extent of cartilage damage. However, our limited amounts of synovial fluid did not allow us to test these markers. Serum concentration assays were possible, but may not have been a completely valid option as Kojima et al. found that synovial fluid proteoglycan concentrations did not reflect reductions of proteoglycan in articular cartilage [41]. Nonetheless, the modified Mankin score has proven to be reliable in characterizing OA progression [42]. Second, we did not record the precise location of synovitis in the knee, which may provide important data regarding the mechanism of synovitis [43]. Third, the PTOA model in this study may have also been exacerbated given underlying idiopathic OA pathology. Given that the study did not include an idiopathic-osteoarthritis-free ACLT group, some of the observed changes may have occurred as a result of natural OA progression in the Guinea pig. To this regard, studies have only identified modest joint disruption at the 5.5-month time point, with the onset of moderate OA associated changes at 12-months of age [44, 45].

Although not detailed in this work, previous mechanistic work has shown that SDF-1 is produced from the synovium to interact, through paracrine signaing, with its receptor CXCR4 on the articular surface. Kanbe et al. showed that SDF-1 is secreted primarily by synovial fibroblasts in models of both osteoarthritis and rheumatoid arthritis at a high level [18]. Further looking at the production of SDF-1, it was shown that in surgical models where the synovium was excised, the levels of SDF-1 in both the synovial fluid and serum dropped precipitously, suggesting that the responsible cells were localized to the synovium [29]. A potential limitation, SDF-1 has also been shown to be produced in subchondral bone marrow [18, 46]. Further research is needed to explore the relative contributions of synovium-derived versus marrow-derived SDF-1, but previous work showing large reductions of SDF-1 concentration after synovectomy without global pharmacologic therapy supports the thesis that the critical volume of SDF-1 originates from the synovium. Furthermore, immunohistochemical methods have been used to localize SDF-1 to the synovial membrane, where SDF-1 positivity was localized in cells lining synovial tissue and in perisynovial epithelial and lymphocytic cells and found to be absent on the articular surface [18, 29].

In summary, this study indicates that the pathogenesis of post-traumatic OA may be synovium mediated and its pathology does not progress uniquely through an SDF-1 related pathway. Future studies are needed to assess other inflammatory pathways related to synovitis that may initiate and maintain post-traumatic OA, and to help differentiate its pathological mechanism from idiopathic OA.

## Conclusions

In this study, it was found that synovial inflammation, independent of cartilage morphology, SDF-1 concentration, and MMP-13 concentration, was markedly different between idiopathic and post-traumatic OA. These results highlight the differing morphological and biochemical profiles of post-traumatic versus idiopathic osteoarthritis and calls for a more thorough examination of the sole of the synovial membrane in the pathogenesis of post-traumatic osteoarthritis.

## Abbreviations

ACLT: Anterior cruciate ligament transection
ANCOVA: Analysis of covariance
ANOVA: Analyses of variance
ELISA: Enzyme-linked immunosorbent assays
ICC: Interclass reliability coefficient
MMP-13: Matrix metalloproteinase-13
OA: Osteoarthritis
RA: Rheumatoid arthritis
SDF-1: Stromal cell-derived factor-1
SF: Synovial fluid
TLRs: Toll-like receptors
